# Enhanced interactions of Kuroshio Extension with tropical Pacific in a changing climate

**DOI:** 10.1038/s41598-021-85582-y

**Published:** 2021-03-18

**Authors:** Youngji Joh, Emanuele Di Lorenzo, Leo Siqueira, Benjamin P. Kirtman

**Affiliations:** 1 School of Earth and Atmospheric Sciences, Georgia Institute of Technology, Atlanta, GA USA; 2 Atmospheric and Oceanic Sciences, Princeton University, Princeton, NJ USA; 3 Rosenstiel School of Marine and Atmospheric Science, University of Miami, Miami, FL USA; 4 Cooperative Institute for Marine and Atmospheric Studies, University of Miami, Miami, FL USA

**Keywords:** Physical oceanography, Climate sciences, Ocean sciences

## Abstract

Quasi-decadal climate of the Kuroshio Extension (KE) is pivotal to understanding the North Pacific coupled ocean–atmosphere dynamics and their predictability. Recent observational studies suggest that extratropical-tropical coupling between the KE and the central tropical Pacific El Niño Southern Oscillation (CP-ENSO) leads to the observed preferred decadal time-scale of Pacific climate variability. By combining reanalysis data with numerical simulations from a high-resolution climate model and a linear inverse model (LIM), we confirm that KE and CP-ENSO dynamics are linked through extratropical-tropical teleconnections. Specifically, the atmospheric response to the KE excites Meridional Modes that energize the CP-ENSO (extratropicstropics), and in turn, CP-ENSO teleconnections energize the extratropical atmospheric forcing of the KE (tropicsextratropics). However, both observations and the model show that the KE/CP-ENSO coupling is non-stationary and has intensified in recent decades after the mid-1980. Given the short length of the observational and climate model record, it is difficult to attribute this shift to anthropogenic forcing. However, using a large-ensemble of the LIM we show that the intensification in the KE/CP-ENSO coupling after the mid-1980 is significant and linked to changes in the KE atmospheric downstream response, which exhibit a stronger imprint on the subtropical winds that excite the Pacific Meridional modes and CP-ENSO.

## Introduction

**Figure 1:**
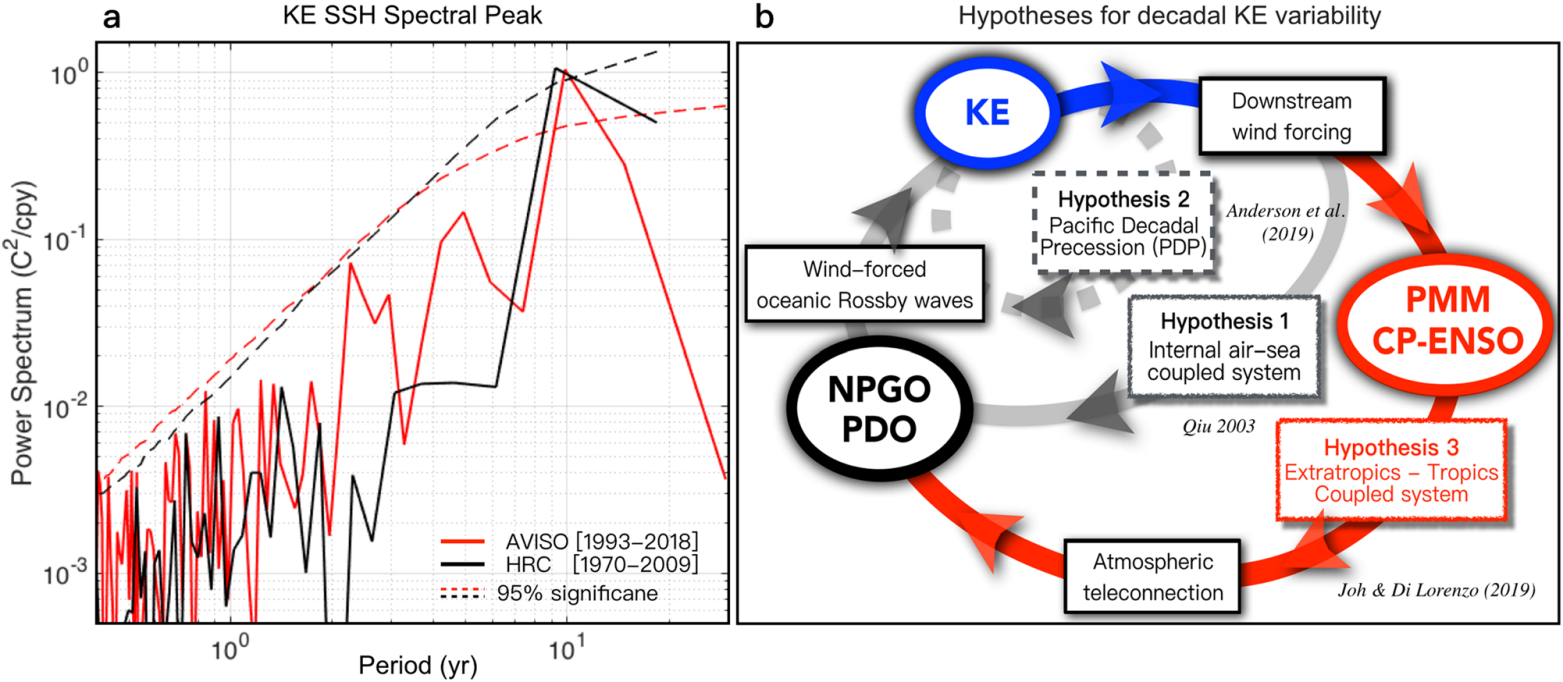
The preferred spectral peak of the KE index and diagram of the proposed hypothesis for generating decadal KE variability. (**a**) Power Spectrum as a function of the SSH-based KE index period (year) from the observations and HRC with 95% significance levels. (**b**) Internal air-sea coupled KE system (KE → KE downstream response → PDO/ NPGO → KE, hypothesis 1^17^), Pacific Decadal Precession KE system (KE → north–south teleconnection phase → east–west teleconnection phase → KE, hypothesis 2^19^, and extratropical-tropical coupled KE system (KE → KE downstream response → PMM/CP-ENSO → NPGO/PDO → KE, hypothesis 3^20^).

The Kuroshio Extension (KE) is a major component of the North Pacific western boundary current (WBC) system and is characterized by prominent decadal fluctuations of sea surface height (SSH) and temperature (SST). These variations are considered to be modulated by mid-latitude air-sea interactions and known for generating and enhancing the decadal to interdecadal variability of the North Pacific coupled ocean–atmosphere system^1–12^. The KE is an *eastward*-flowing *oceanic jet* accompanied by large amplitude meanders, a high level of mesoscale eddy variability, and linked to marked features of ocean circulation dynamics over the North Pacific^13–16^. The broad but significant spectral peak of KE SSH fluctuations (~ 10 years, Fig. 1a) illustrates the substantial decadal variability of the North Pacific climate^3,17,18^; thus, it is essential to identify the mechanisms behind the preferred decadal timescale of KE dynamic system.

Several hypotheses, based on empirical statistical analysis, have been proposed to explain the preferred decadal peak of KE variability (Fig. 1b). Qiu^17^ has described the KE as an internal North Pacific air-sea coupled system, where the delayed atmospheric response of the downstream region can drive the wind-forced SSH signals over the central/eastern North Pacific. (Hypothesis 1 in Fig. 1b). In Qiu’s hypothesis, the KE downstream wind response projects onto the atmospheric forcing of KE, which excites the oceanic Rossby waves that propagate the signal back into the ocean western boundary switching the decadal KE phase. Follow-up studies demonstrated that KE's negative feedback could enhance the KE predictability on the decadal timescale by using a two-way wave adjustment plus wind feedback scenario^17,21^.

On the other hand, Anderson^19^ has linked the decadal KE dynamics to a new mode of quasi-decadal variability, the Pacific Decadal Precession (PDP)^22,23^, which is characterized by a ~ 10 yr counterclockwise progression of a dipole structure of the North Pacific atmosphere (Hypothesis 2 in Fig. 1b). This hypothesis asserts that the evolution of the PDP mirrors a quasi-decadal signal in KE. The mesoscale KE variations are dynamically linked to the progression of large-scale atmospheric circulations through changes in zonal wind stress anomalies in the subpolar frontal zone (SPFZ). In hypothesis 2, the KE-PDP dynamics are represented as the migration of subsurface temperature and underlying meridional surface dipole circulations from the eastern to the western North Pacific. In contrast, in hypothesis 1, the midlatitude wind anomalies are described as the direct forcing of KE that triggers the westward propagating oceanic Rossby waves^24–30^. More recently, Siqueira et al.^31^ suggest, based on observational estimates and ocean eddy-resolving coupled retrospective forecasts, that the near-decadal variability associated with PDP can lead to wind-forced SSH signals that affect the KE state with a time lag of about four years. They investigate the downstream atmospheric and oceanic response to the KE, showing that the dipole in wind stress curl anomalies in the eastern North Pacific does not effectively project on the forcing pattern for the KE variability but is more consistent with the meridional mode forcing by weakening the trade winds in the subtropical eastern Pacific^20^.

**Figure 2:**
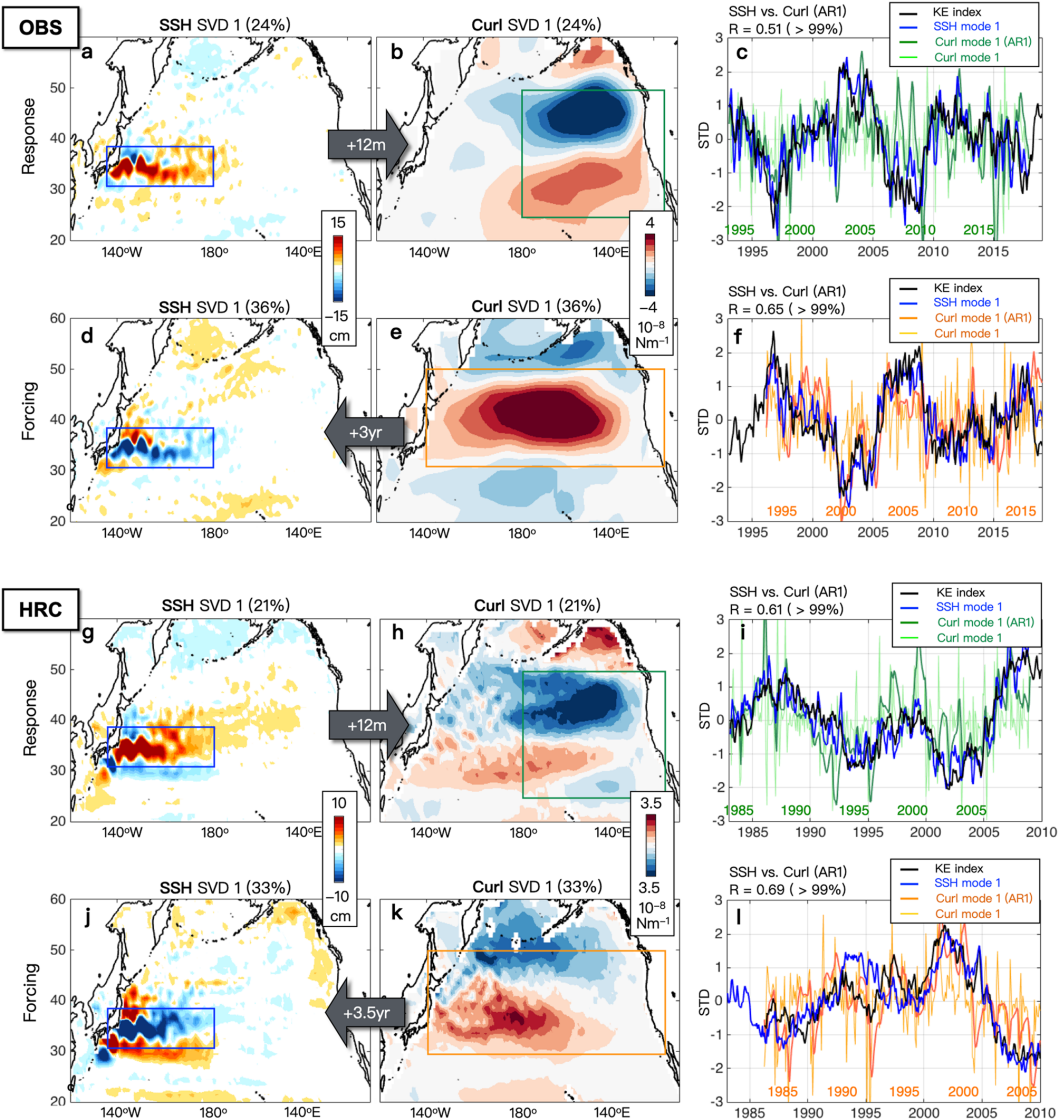
Spatial and temporal patterns of KE dynamics (KE atmospheric response and forcing of KE) based on singular value decomposition (SVD) analysis between KE SSH and midlatitude wind stress curl with lead/lag times. Patterns of the leading SVD mode between the observed (**a**) SSH over the KE region (blue box) and (**b**) 12mon-lagged Curl over the downstream region (green box) for the recent period. (**c**) The associated time series of corresponding SSH and SVD modes. (d-f) Same as in (**a**–**f**), but for with mon-leading Curl. The same analysis was repeated with HRC in (**g**–**i**) and (**j**–**l**). The figures are provided using MATLAB R2017a (https://www.mathworks.com/products/new_products/release2017a.html).

While these two proposed hypotheses (Hypothesis 1 and 2 in Fig. 1b) describe KE as the internal air-sea coupled system within the North Pacific, Joh and Di Lorenzo^20^ provide observational evidence showing that the preferred decadal timescale of KE may arise from the interaction between KE and central tropical Pacific (CP) variability, especially El Niño Southern Oscillation (ENSO) through Pacific Meridional Modes (PMM) (Hypothesis 3 in Fig. 1b). Through the available observations, this third hypothesis argues that the downstream atmospheric feedback can be interpreted as a slow nudging of the storm track (e.g., wind stress curl, 0–12 months timescale) that projects onto the atmospheric forcing of PMM and CP-ENSO. Specifically, Joh and Di Lorenzo^20^ have demonstrated that the persistent KE downstream wind feedback associated with northward migration of the extratropical storm track projects on the atmospheric forcing pattern of the North Pacific Oscillation (NPO), which in turn is known to initiate the North PMM through reducing wind-evaporation feedback^7,32,33^. Based on the persistent KE downstream response from 0 to 12 months reported by previous studies (see Fig. 2 in Joh and Di Lorenzo^20^), such as the low sea level pressure anomalies^31^, positive wind stress curl anomalies^5^, and positive Ekman pumping velocity field anomalies^21^ over the subtropical Northeast Pacific (170°–130° E & 20°–40° N), Joh and Di Lorenzo^20^ have explained that this surface cyclonic atmospheric response (e.g., the southern lobe of NPO) opposes the mean trade winds and produces strong downward latent heat flux anomalies that grow the warm SST resembling PMM-like oceanic signatures. They note that once the KE downstream wind response contributes to the atmospheric forcing of the PMM^7,20,32,33^, the propagation of the PMM anomalies favors the initiation of ENSO in the tropics (*also with a possible contribution of Trade wind charging effect*^34–36^). During ENSO, the tropical convective system excites the atmospheric teleconnection projecting the tropical signal back into the North Pacific; thus, corresponding changes in the surface heat fluxes and wind-driven mixing associated with the oceanic forcing of KE (e.g., westward propagating Rossby waves) are followed^37,38^. By pointing out a significant difference in the observed spatial patterns of the atmospheric response and forcing of KE, the study hypothesizes that the ENSO atmospheric teleconnections play a key role in enhancing and evolving the KE downstream wind feedback into the large-scale wind forcing, which drives the SSH anomalies that excite the westward propagating Rossby waves^24–30^.

Consistent with the above hypothesis, the Joh and Di Lorenzo^20^ work shows that the lead-lag correlation between the KE and the PMM/CP-ENSO indices exhibits a significant sinusoidal shape, where the interval between their correlation peaks correspond to the 10 years. Based on spatial and temporal evolutions of the KE variability using the observational data, Joh and Di Lorenzo^20^ suggest that KE might significantly covary with the tropics on decadal timescales, giving rise to the preferred spectral peak of KE. To provide further support for this observed extratropical-tropical coupled KE system; however, additional analyses using numerical models and large number realizations of the climate system are required.

The objective of this study is to expand upon the observational findings reported in Joh and Di Lorenzo^20^ by investigating the extratropical-tropical coupled KE system represented in the numerical simulations (e.g., high-resolution (0.1°) coupled model, HRC) and empirical model ensembles (Linear Inverse Models, LIM). In the sections that follow, we first show strong agreement between the observations and HRC on the KE dynamics (e.g., time-scale, atmospheric response, and forcing of KE) and its coupling to the central tropical Pacific. We also report on a consistent change in the interactions of KE and CP-ENSO under a warmer climate. To assess the significance of these changes, we compare KE's decadal statistics before and after the mid-1980 by using reconstructed indices of KE and CP-ENSO in LIM ensembles. Describing the statistical changes in the KE dynamics system, we provide a discussion of the detection and attribution of significant changes in the decadal KE dynamics in a changing climate.

## Results

### Observed and simulated dynamics of KE variability

We first conduct a singular value decomposition (SVD) analysis to examine the KE atmospheric downstream response and atmospheric forcing of KE based on previous studies that documented the air-sea coupled KE system^3,5,20,21,31^. The SVD analysis identifies the coupled relationship between the KE SSH and mid-latitude wind anomalies as the response/forcing feedback by extracting the maximum covariability of two variables (e.g., SSH and wind stress curl)^39,40^. To identify the KE atmospheric response (KE ocean atmosphere) and forcing (e.g. atmosphere KE ocean) patterns, we apply the SVD to the covariance matrix between the KE SSH anomalies [31°–36° N & 140°–165° E] and large-scale wind stress curl anomalies using different lead (e.g., forcing pattern) and lag (e.g., response pattern) relationships. For example, we use *1 yr lagged* Northeast Pacific wind stress curl [25°–50° N & 180°–120° W] for identifying the KE atmospheric response (Fig. 2a,b), which is associate with a shift in extratropical storm tracks^21^. In contrast, to extract the atmospheric forcing of KE (Fig. 2d,e), we use *3 yr leading* mid-latitude wind stress curl [30°–50° N & 140°E–120° W], which shows an active center of action in Ekman pumping on the latitudes of the KE where the westward-propagating Rossby waves are excited^3,21,41–44^.

The leading SSH SVD mode clearly represents the well-known spatial patterns of KE variability (Fig. 2a,d), and their time series are consistent with the original KE index (area-averaged SSHa in the KE region) (Fig. 2c,f)^21^. We note that in both the observations and HRC, the Curl SVD patterns reveal a clear difference in their spatial structures between the atmospheric response and forcing of KE (Fig. 2b,h vs. d,k). Specifically, while the KE downstream wind stress response shows a dipole structure of curl anomalies limited in the Northeast Pacific region (Fig. 2b,h), the wind forcing of the KE rather exhibits the large-scale wind anomalies over the central North Pacific (Fig. 2d,k). A discrepancy of spatial patterns of (leading) Curl SVD mode between the observations (Fig. 2a) and HRC (Fig. 2k), where the center of curl anomalies is shifted toward the west in HRC, is considered as model bias related to the pathway of the baroclinic Rossby waves between the midlatitude and western boundary over the Pacific region.

We find that the SVD analysis is consistent with the observational findings reported by Joh and Di Lorenzo^20^ over the period 1980-present and HRC also captures the lead/lag relationships between KE and CP-ENSO. For example, correlation maps between the KE index and lagged SST/Curl/SSH anomalies from HRC (Supplementary Figures S1) support that (1) the KE atmospheric downstream feedback can evolve into the atmospheric forcing (e.g., the NPO-like dipole structure of downstream wind stress curl in the black box of Figure S1-b, at lag 12–24 months; *consistent with Fig. **2** in Joh and Di Lorenzo*^20^) that projects PMM and CP-ENSO (Figures S1-a, at lag 36–60 months) and (2) the resulting atmospheric teleconnections from the warm tropical SST enhance the North Pacific atmospheric anomalies (e.g., North Pacific low pressure (positive wind stress curl) associated with the Pacific-North America (PNA) pattern, Figures S1-b at lag 48 and 60 months) that drives the oceanic Rossby waves (Figures S1-c, at lag 60 months)^37,38^. The strong development of warm tropical SST (Figures S1-a) and the corresponding robust SSH anomalies over the central North Pacific (Figures S1-c) at lag 60 months indicate that tropical processes affect the evolution of the KE system (e.g., oceanic baroclinic adjustment forced by atmospheric teleconnections)^37,38^. While the SVD mode is a statistical mode, which might not perfectly capture dynamical relationships, we confirm a good agreement in the spatial patterns between the SVD results (Fig. 2) and the response/forcing of KE shown in the KE regression patterns (Figures S1). Overall, the coupled climate model simulation captures the observed interactions between the extratropical KE and tropical Pacific and tracks the spatial–temporal transitions of the KE related to its preferred decadal time scale. The statistical significance of these relations in both the model and observations is further explored in the next sections.

### Non-stationarity in the relationship between KE and CP-ENSO

**Figure 3:**
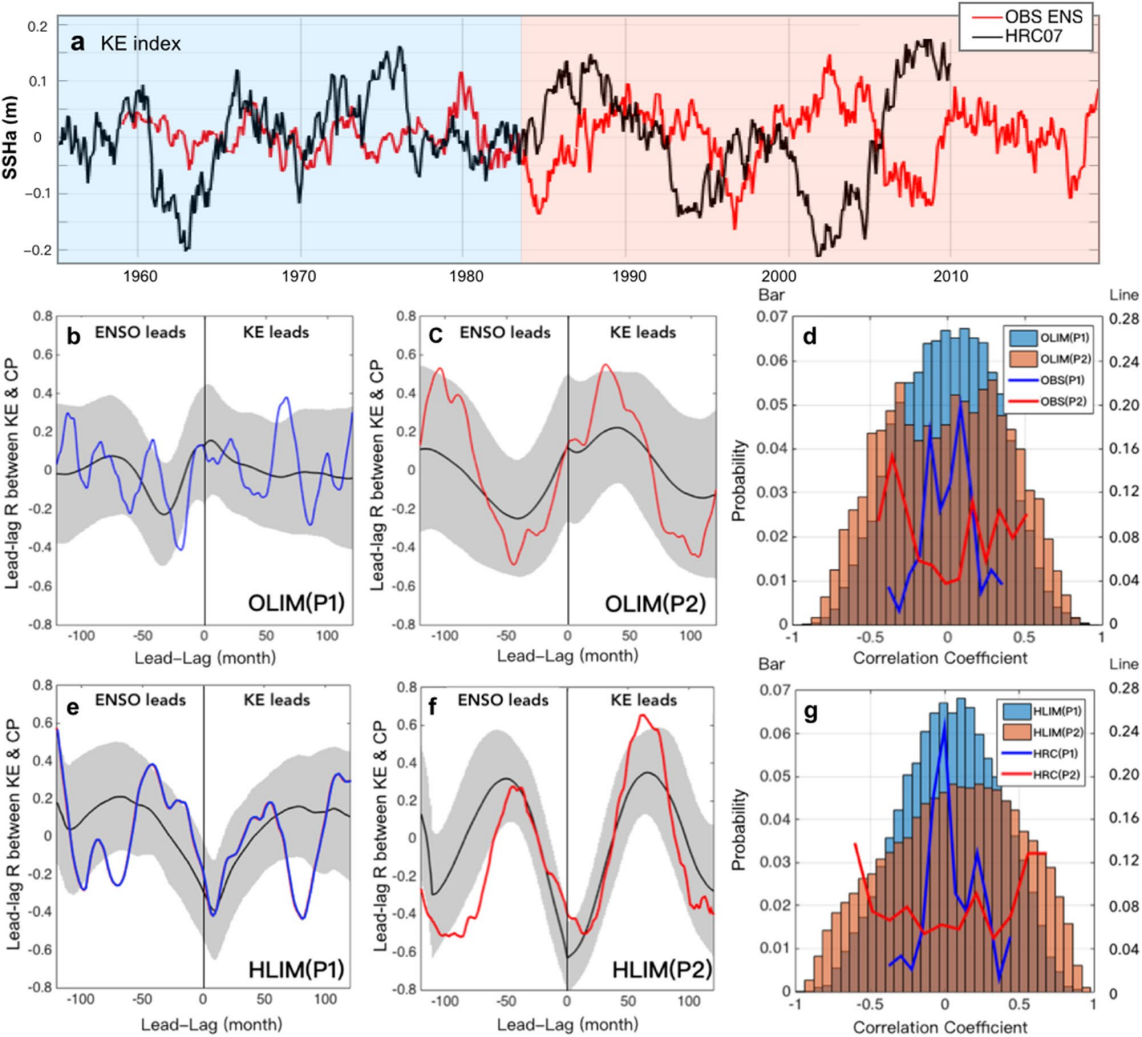
Changes in the temporal KE variability and coupling between the KE and CP-ENSO from Period 1 to Period 2. (**a**) Computed KE index using area-averaged SSH anomalies over the KE region from the observational ensembles (ORA and AVISO, red line) and HRC (historical run-no assimilation, black line). Lead-lag correlation between the KE and CP-ENSO indices during the (**b**) P1 and (**c**) P2 in LIM constructed by observational SSH and SST (OLIM), where black and blue/red lines are each the ensemble mean, and the observed correlation and shadings show plus and minus two standard deviations in the mean values. (**d**) Probability distribution function of the observed (lines) and reconstructed (bars) correlation coefficients of the panel b and c for the P1 and P2 respectively. Panels (**e**, **f**, and **g**) are same as (**b**, **c**, and **d**), but for LIM constructed by HRC SSH and SST (HLIM).

Given a substantial agreement between the observed and simulated dynamics of the KE, we note some changes in characteristics of the KE variability during the recent decades after the 1980s. We note that strong decadal fluctuations of KE with the preferred spectral peak ~ 10 year only appear after the mid-1980 (Fig. 3a, *note that HRC is not a retrospective forecast, but a free-running simulation with no assimilation*) (see also wavelet analysis Figure S3). We suggest that the intensification of the KE decadal variance is linked to the strengthening of the interaction between KE and CP-ENSO, which appears to be non-stationary. The intensification of the interaction between KE and CP-ENSO is evident from comparing the cross-correlation function between KE and CP-ENSO in the period 1960–1984 (a blue line in Fig. 3b), which shows no significant periodicities, and the period 1985–2019 (a red line Fig. 3c), which is characterized by a sinusoidal shape with the period of 10 years. This change in relationships between KE and CP-ENSO is also captured in the climate model simulations (Fig. 3e,f, compare blue and red lines, respectively) and is evident in the spatial evolution of the KE atmospheric/oceanic response after the mid-1980 in HRC (Figure S1), which shows a clear transition from the KE state to the PMM/CP-ENSO mode with the stronger amplitudes compared to those during earlier decades (Figures S2).

To assess whether the above temporal differences in characteristics of KE system are a matter of chance or are statistically significant, we employ a Linear Inverse Model (LIM), which can extract dynamical properties of the climate system from its observed behaviors and statistics^45–50^. Linear inverse modeling describes the relevant dynamics as the form of a linear stochastic differential equation that consists of the state of the system, the evolution operator, and the stationary white noise. The LIM approach has been successfully used to reconstruct the optimal growth of the major climate variability and extract optimal forcing patterns. For example, LIM captures the observed evolution of different types of ENSO events^45^, detailed processes of ENSO dynamical feedback^46^, and also shows a potential for the skillful prediction of Pacific Decadal Oscillation^50^.

Here we construct LIMs based on a reduced space of empirical orthogonal functions (EOFs) with the corresponding principal components (PCs) as the state vectors. To examine the interaction between KE and CP-ENSO in LIM, we define the state vectors using the basin-scale SSH [31°–36° N & 140°–165° E] and SST [20° S–60° N & 120° E–80° W] anomalies to reconstruct the KE and CP-ENSO indices. In this study, the CP-ENSO index was defined by employing two principal components of tropical Pacific SSTa, whose spatial signature is constrained to the central Pacific anomalies^51^. We retain 8–15 leading EOFs (vary depending on different LIM sets), explaining more than 70% of the total variance. To compare the KE variability between before and after the mid-1980, we set two 25-year time-periods as 1960–1984 (previous period, P1) and 1985–2019 (recent period, P2), then construct LIMs separately for P1 and P2. To build each LIM set, we first determine the system dynamics and stochastic forcing, which capture the observed spatial/temporal characteristics of KE and CP-ENSO for each P1 and P2 (Supplementary Figures S4). Next, we integrate each period of LIM for 25,000 years, respectively, following the method in Penland and Matrosov^47^, then we divide the output into one thousand 25-year segments as the large ensembles for each P1 and P2. Those one thousand 25-year segments are used to estimate the sampling uncertainties for the statistical significance test. We repeat the same LIM construction using HRC, but with 20-year time-periods of P1 (1960–1979) and P2 (1980–2009) (Supplementary Figures S5). We hereafter refer to LIMs with the different dataset as *OLIM* for LIM using observations and *HLIM* for LIM using HRC and define each period of OLIM and HLIM as *OLIM(P1)* and *HLIM(P1)* for the *earlier period (P1)* and *OLIM(P2)* and *HLIM(P2)* for the *recent period (P2).* We use the output of these several different LIMs to investigate and compare the KE dynamic between the two periods.

A comparison of the lead-lag correlations of KE and CP-ENSO indices between OLIM(P1) vs. OLIM(P2) and HLIM(P1) vs. HLIM(P2) indicates apparent differences in the relationship between KE and the tropics for the two different periods (black lines in Fig. 3b,c,e,f). While OLIM(P2) and HLIM(P2) show a significant sinusoidal shape of correlation function distribution (Figs. 3c,f) similar to the observed, OLIM(P1) and HLIM(P1) exhibit a nearly even distribution (Fig. 3b,e). We note that the time interval of recurring peaks in the lead-lag correlation of OLIM(P2) and HLIM(P2) is consistent with the observed interval, which corresponds to ~ 10 years (Fig. 3c).

A power spectrum analysis of the KE index shows that the quasi-decadal peak corresponding to 10 years is dominant in both the OLIM(P2) and HLIM(P2) (Supplementary Figures S6). We suggest that this preferred KE spectral peak during P2 may arise from the decadal interaction with the tropical Pacific. The lead-lag correlation sampling distribution of KE and CP-ENSO between P1 and P2 (Fig. 3d) shows a noticeable difference in shape, confirming the change in the occurrences of lead-lag correlation values. For example, the observed lead-lag correlations between the KE and CP-ENSO indices (lines in Fig. 3d) and the probability density functions of the reconstructed correlation in OLIMs (bars in Fig. 3d) show that OLIM(P2) has a heavy-tailed distribution. This tail-heavy distribution indicates that higher correlations (i.e., stronger coupling) are more frequent in P2. We find similar results when we repeat the same analysis using HLIMs (Fig. 3e–g). Combining the consistent results from OLIMs (Fig. 3b–d) and HLIMs (Fig. 3e–g), we suggest that the KE variability between P1 and P2 is different from each other (i.e., we reject the null hypothesis that the differences are merely sampling issues). Specifically, from the LIM ensemble modeling, we conclude that the statistics of KE in P1 (e.g., OLIM(P1) and HLIM(P1)) are significantly different from those in P2 (e.g., OLIM(P2) and HLIM(P2)) (e.g., sampling distribution), suggesting that the KE variability is non-stationary over the period 1958–2009. This allows us to reject the null hypothesis that these changes arise from random sampling of the same dynamical operator.

Before concluding, however, we note that the correspondence between model and observations in capturing a significant change in the relationship between KE and CP-ENSO after the 1980s requires further investigation into what is the shared external forcing mechanism that leads to these changes in model and observations (e.g., climate change). We also note a non-negligible difference between the observations and HRC regarding the time-scale of their lead-lag correlations during P2 (compare Fig. 3c vs. f.). For example, in the observations, KE is accompanied by the neutral phase of ENSO at lag 0 years and drives CP-ENSO at lag ~ 3 years (Fig. 3c), whereas in HRC, KE is concurrently in the La Niña phase and induces CP-ENSO at lag ~ 5 years (Fig. 3f). Therefore, the question is whether this difference is associated with a variation in LIM realizations or should be considered a model error in HRC. Because the LIM’s dynamical operator is fixed, the ensemble spread results only from different initial conditions, which is comparable to internally generated noise forcing^52^. In nature, this atmospheric noise forcing can generate not only interannual fluctuations but also lower frequency variations by stochastic excitation of climate variability and following red noise process (e.g., PMM and ENSO^53–56^). Thus, identifying the relative importance of stochastic forcing in the decadal KE variability is essential to assess the KE dynamic system's uncertainty and predictability. In the next section, we examine the role of this noise component in LIM, which is intrinsically generated in the stochastic differential equation, and investigate the LIM ensemble spread in the coupling between KE and CP-ENSO.

### Role of stochastic forcing on decadal KE dynamics

**Figure 4:**
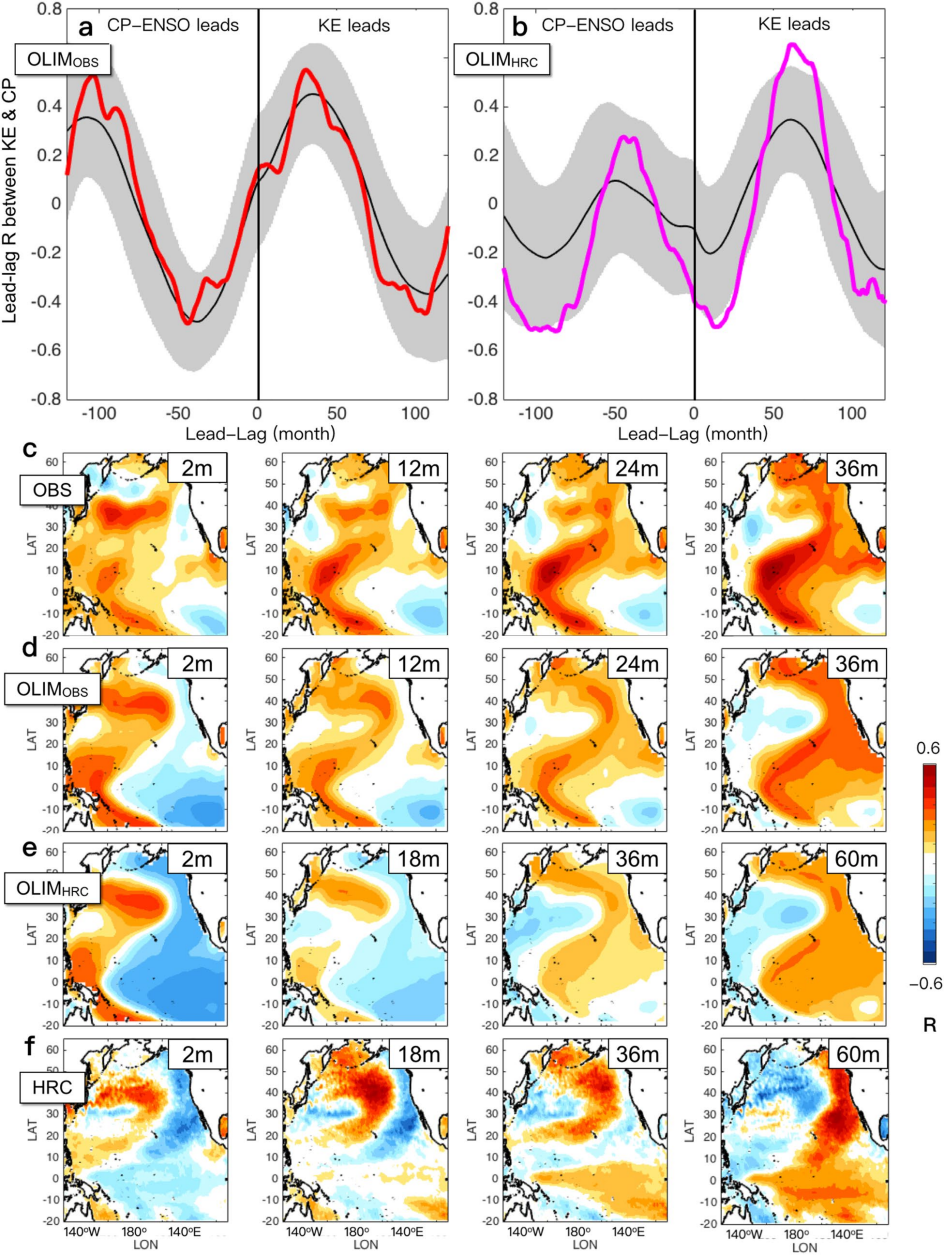
Variation of the extratropical-tropical KE system in the presence of stochastic noise forcing. 50-member subsets of OLIM(P2) that best fit to the (**a**) observed (OLIM_OBS_) and (**b**) simulated (OLIM_HRC_) lead-lag correlation between the KE and CP-ENSO indices, where ensemble means and references are denoted as black and red/pink lines with the two standard-deviation confidence interval (shading) in OLIM_OBS_ and OLIM_HRC_ respectively. The dot indicates the grid whose signal is above 95% significance level based on the student t-test. SST progression of KE in the (**c**) observations, (**d**) OLIM_OBS_, (**e**) OLIM_HRC_, and (**f**) OLIM_HRC_. The figures are provided using MATLAB R2017a (https://www.mathworks.com/products/new_products/release2017a.html).

Comparing across the individual realization in LIMs allows for understanding how the observed extratropical-tropical KE system can vary in the same dynamical system (e.g., the fixed dynamical operator of LIM). In other words, can the phase difference in the KE/CP-ENSO coupling between the observations and HRC shown in Fig. 3c,f be captured within the same dynamical operator? To investigate whether the same dynamical operator of OLIM(P2) can reproduce both the observed and simulated characteristics of the lead-lag correlations of KE and CP-ENSO, we subsample two sets of 50 members out of 1000 members of OLIM(P2) whose correlations are the best fit to each observed (redlined in Fig. 3c) and simulated (redlined in Fig. 3f) lead-lag correlations, as OLIM_OBS_ and OLIM_HRC_ respectively. To find the subset OLIM_OBS_ (OLIM_HRC_), we compute the correlation of the observed (simulated) and reconstructed lead-lag correlation between the KE and CP-ENSO indices in each member of OLIM(P2), and select the top 50 members based on the computed correlations and show the resulting subset of lead-lag correlations between the KE and CP-ENSO indices in Fig. 4a (Fig. 4b). Because OLIM(P2) is constructed based on observational estimates of SSH and SST anomalies to capture the observed characteristics of KE, it is not surprising that the ensemble spread of OLIM_HRC_ is much larger compared to that of OLIM_OBS_ and do not fit as well as in OLIM(P2). However, we note that some portion of the realizations of the observational LIM (e.g., OLIM_HRC_) can also capture the simulated lead-lag correlations between KE and CP-ENSO that significantly overlap with HRC (Figs. 4b).

Conversely, using HLIM subsets (Supplementary Figs. 7c and 7d), we find similar results revealing that the dynamic system of HLIM can also represent both different types of coupled KE system shown in the observations and HRC during P2. Taken together, we suggest that the difference in the lead-lag correlation of KE and CP-ENSO between the observations and HRC (Figs. 4b vs. e) might be due to the sampling and that the KE behaviors can be irregular and dynamically vary depending on the noise component in the climate system. Simply put, the differences between P1 and P2 are not sampling and are attributed to changes in the KE system, whereas the difference between HRC and observational estimates may indeed be due to sampling.

**Figure 5:**
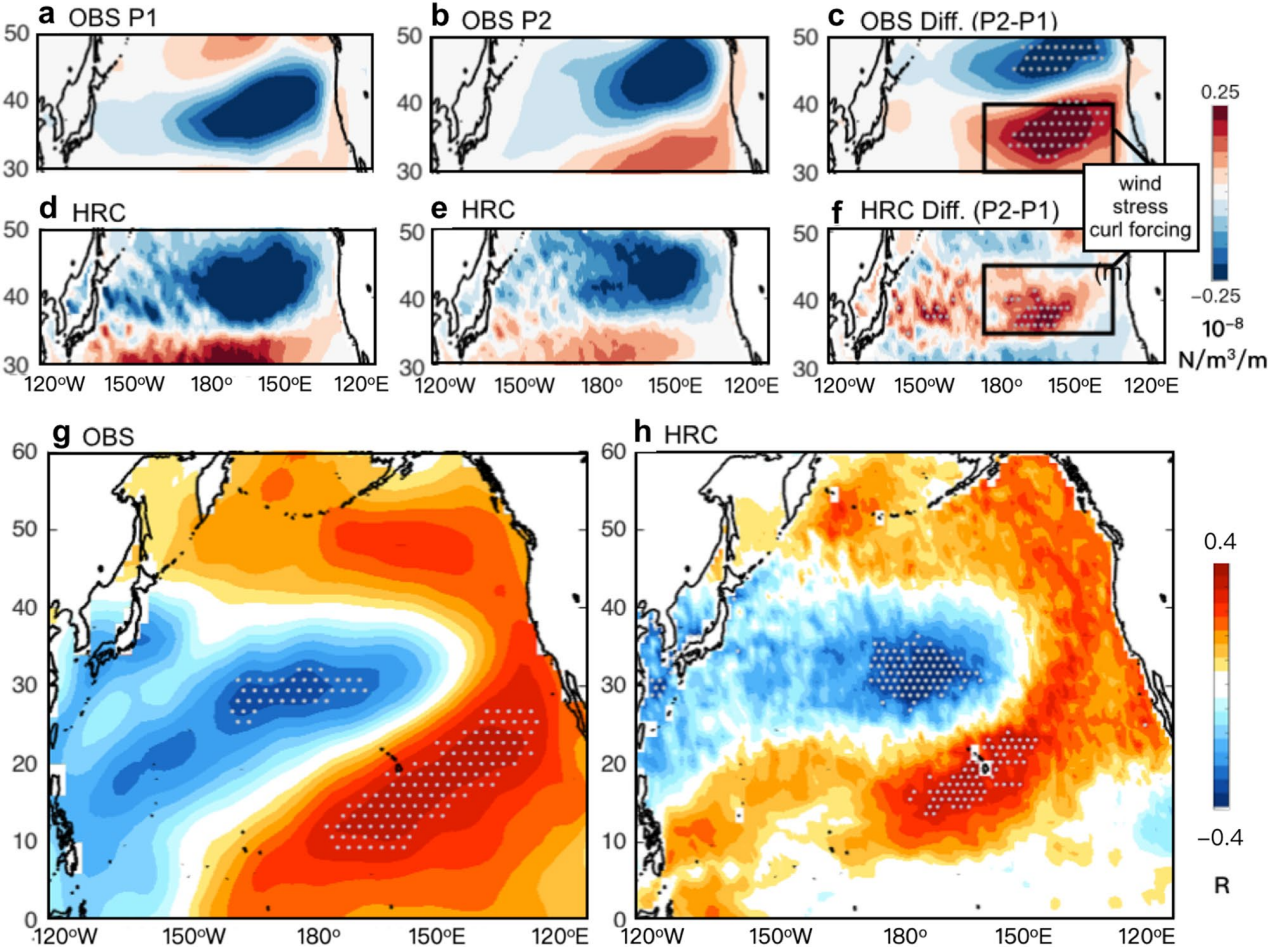
Observed and simulated changes in the KE atmospheric downstream response from the P1 to P2 Patterns of leading Curl SVD mode between KE SSH and midlatitude Curl (12mon-lagged) for the (**a**) Period 1 and (**b**) Period 2 and its difference (**c**) in observations. (**d**–**f**) Same as (**a**–**c**), but for in HRC. (**g**, **h**) Correlation map of SST anomalies with the time series of subtropical wind forcing (black box of **d** and **g**) that is computed by regressing the target patterns (black box of **c** and **f**) onto the original wind stress curl anomalies. The figures are provided using MATLAB R2017a (https://www.mathworks.com/products/new_products/release2017a.html).

Supporting the above findings that the difference in the phase of KE/CP-ENSO coupling falls within the sampling distribution, which can happen in the same dynamic system, the spatial evolutions of KE oceanic response from OLIM_OBS_ (Fig. 5d) and OLIM_HRC_ (Fig. 5e) exhibit the similar progression of SST (KE oceanic response). Both OLIM_OBS_ (Fig. 5d) and OLIM_HRC_ (Fig. 5e) show that the KE SST signal becomes the warm anomalies in the Northeast Pacific resembling the PMM-like oceanic signature and induce ENSO-like SST warming in the tropics as seen in the entire ensemble mean of OLIM (Supplementary Figure S8). Although there is an apparent temporal difference of the KE SST progression between the observations (KE CP-ENSO (~ 3 years) in Fig. 4c,d) and HRC (KE CP-ENSO (~ 5 years) in Fig. 4e,f), this might be associated with variation in the internal variability (e.g., stochastic noise forcing) as well as the concurrent KE-ENSO that can affect a transition from KE to ENSO. Specifically, while in the observations and OLIM_OBS_, the KE SST at lag 2 months (Fig. 4c) exhibits the west–east dipole SST anomalies over the tropics with the eastern Pacific La Niña signature, in OLIM_HRC_ and HRC (Fig. 4d–f), KE is accompanied by the broad cold SST anomalies extending from the west Pacific warm pool to the South American coast, resembling the central Pacific La Niña mode. Depending on which tropical SST mode is accompanied by KE and how the different mean background affects the transition time-scale from KE to PMM/ENSO, the temporal interaction between KE and CP-ENSO can vary. The overall similarity between OLIM_OBS_ and OLIM_HRC_ in the KE SST progression supports the argument that the observations and HRC can be considered as any individual ensemble member generated in the same KE dynamical system.

## Discussion

Through complementary analysis using observations, numerical simulations, and empirical dynamical models, this study explores the differences in the KE properties between before and after the mid-1980 (e.g., P1 vs. P2). Having shown that the decadal interaction between KE and CP-ENSO has become more robust in the recent period, we propose that the KE variability is not stationary and that these recent changes in the KE system may reflect a response to a changing climate. We first note the contributions of changes in the background mean state (e.g., surface winds) KE system. A comparison of the KE downstream atmospheric response between P1 and P2 (Fig. 5a/d vs. b/e) shows that the dipole structure of wind stress curl over the Northeast Pacific has shifted towards the pole, and drives significantly stronger wind forcing in the KE band (31°–39° N) during P2 (Fig. 5c,f). As many previous studies have described, this subtropical wind forcing is linked to positive air-sea thermodynamic coupling known as the winds-evaporation-SST (WES) feedback^57^; thus, we suggest that the spatial shift in KE wind stress response pattern may induce CP-ENSO activity by preferentially projecting stronger PMM-like oceanic signatures onto the SST in the recent period. This is evident by the strong PMM footprint that emerges in the correlation map of SST anomalies (Fig. 5g,h) with the time series of subtropical wind forcing (black boxes Fig. 5c,f) that is computed by regressing the target patterns (black boxes Fig. 5c,f) onto the original wind stress curl anomalies.

Consistent with the above findings, we also detect noticeable changes in the climatology of the surface winds (e.g., zonal wind), showing that the subtropical westerly winds have been significantly intensified after mid-1980, especially for the spring season (Supplementary Figure S9). Considering that the mid-latitude stochastic atmospheric forcing can affect the ENSO via the seasonal footprinting mechanism^33^, it is plausible that the changes in the background climate might be dynamically linked to the enhanced interaction between KE and CP-ENSO by altering the KE downstream atmospheric response (e.g., the latitudinal position of extratropical storm track). On the other hand, we cannot exclude the external forcing on the coupled KE dynamics and related background state, because previous studies have shown that the extratropical-tropical coupling is intensifying under anthropogenic forcing^58–62^. Specifically, the increasing link between the North Pacific ENSO precursor (e.g., North Pacific Oscillation) and ENSO under the enhanced greenhouse forcing^62,63^ might be associated with the stronger coupling between KE and CP-ENSO. Therefore, how and how much the background mean state and the external forcing independently or/and jointly affect the decadal KE system should be further investigated through appropriate modeling experiments (e.g., pre-industrial control vs. historical forcing).

By diagnosing KE simulations in climate models, this study provides an understanding of the KE dynamics' underlying mechanism concerning the extratropical-tropical coupling of the Pacific climate. As the KE is the dominant mode of Pacific SSH that gives an enormous impact on the low-frequency variance of biogeochemical quantities and marine ecosystem over the Pacific region, the increasing decadal fluctuations between KE and CP-ENSO may lead to a better quantification of practical predictive skill, uncertainties, and regional impacts of various components of the Pacific decadal variability^43,64^.

## Data and method

### Model

This study uses monthly mean data of SSH, SST and wind stress curl from National Center for Atmospheric Research (NCAR) Community Climate System Model Version 4 (CCSM4) current-day climate simulation, which is composed of the Community Land Model (CLM), the Community Atmospheric Model (CAM), the Los Alamos Parallel Ocean Program (POP) ocean general circulation model, and the Community Ice Code (CICE) with exchanging the state information and fluxes via a coupler^65^. We use a historical run, where the observational estimates of external forcing from 1941 to 2009 are employed in CAM-4.0 atmospheric model configured to 0.5° × 0.625° latitude/longitude grid. Specifically, following the climate of the twentieth Century protocol of the Coupled Model Intercomparison Project version 5 (CMIP5)^66^, the changing historical CO2 fluxes are applied at the air-sea interface are calculated at (6-h) intervals using state variables from the atmospheric model linearly interpolated onto the oceanic grid. The POP2 ocean model uses a tripolar ocean grid^67^ with a ~ 0.1° resolution that allows eddy formation and evolution^67,68^.

### Data

Observations for investigating the SSH-based KE variability are obtained by two different SSH dataset within the period between 1959 and 2018 from the European Centre for Medium-Range Weather Forecasts (ECMWF) Ocean Reanalysis System: ORA-S3 [1959–2009]^69^, Simple Ocean Data Assimilation (SODA) reanalysis version 3^70^, and satellite distributed by Archiving, Validation, and Interpretation of Satellite Oceanographic (AVISO) data [1993–2018] obtained from https://www.aviso.altimetry.fr/en/data/products/auxiliary-products/mss.html. To explore the KE variability at the air–sea interface, we use the monthly mean SST data [2° × 2°horizontal grid] of the National Oceanic and Atmospheric Administration Extended Reconstruction SST, version 3 (ERSST.v3) product^71^ and zonal and meridional wind stress from European Centre for Medium-Range Weather Forecasts (ERA) reanalysis product^ 72^. All anomalies are constructed by removing the mean monthly climatology and linear trend at each grid point.

## Supplementary Information

Supplementary Figures
