# Supplementary material for: Enhanced interactions of Kuroshio Extension with tropical Pacific in a changing climate
Source: Sci Rep. 2021 Mar 18;11:6247. doi: 10.1038/s41598-021-85582-y (PMC7973761; doi:10.1038/s41598-021-85582-y)
Supplement: Supplementary file 1 — Supplementary Figures [file 41598_2021_85582_MOESM1_ESM.docx]

**Supplementary information for**

**Enhanced interactions of Kuroshio Extension with tropical Pacific in a changing climate**

**Scientific Reports**

Youngji Joh^1,2*^, Emanuele Di Lorenzo^1^, Leo Siqueira^3^, and Benjamin P. Kirtman^3,4^

^1^School of Earth and Atmospheric Sciences, Georgia Institute of Technology, Georgia, USA

^2^Atmospheric and Oceanic Sciences, Princeton University, Princeton, New Jersey, USA

^3^University of Miami, Rosenstiel School of Marine and Atmospheric Science, Florida, USA

^4^University of Miami, Cooperative Institute for Marine and Atmospheric Studies, Florida, USA

*Corresponding author (youngji.joh@princeton.edu)

**Contents of this file**

Figures S1-S9.

**
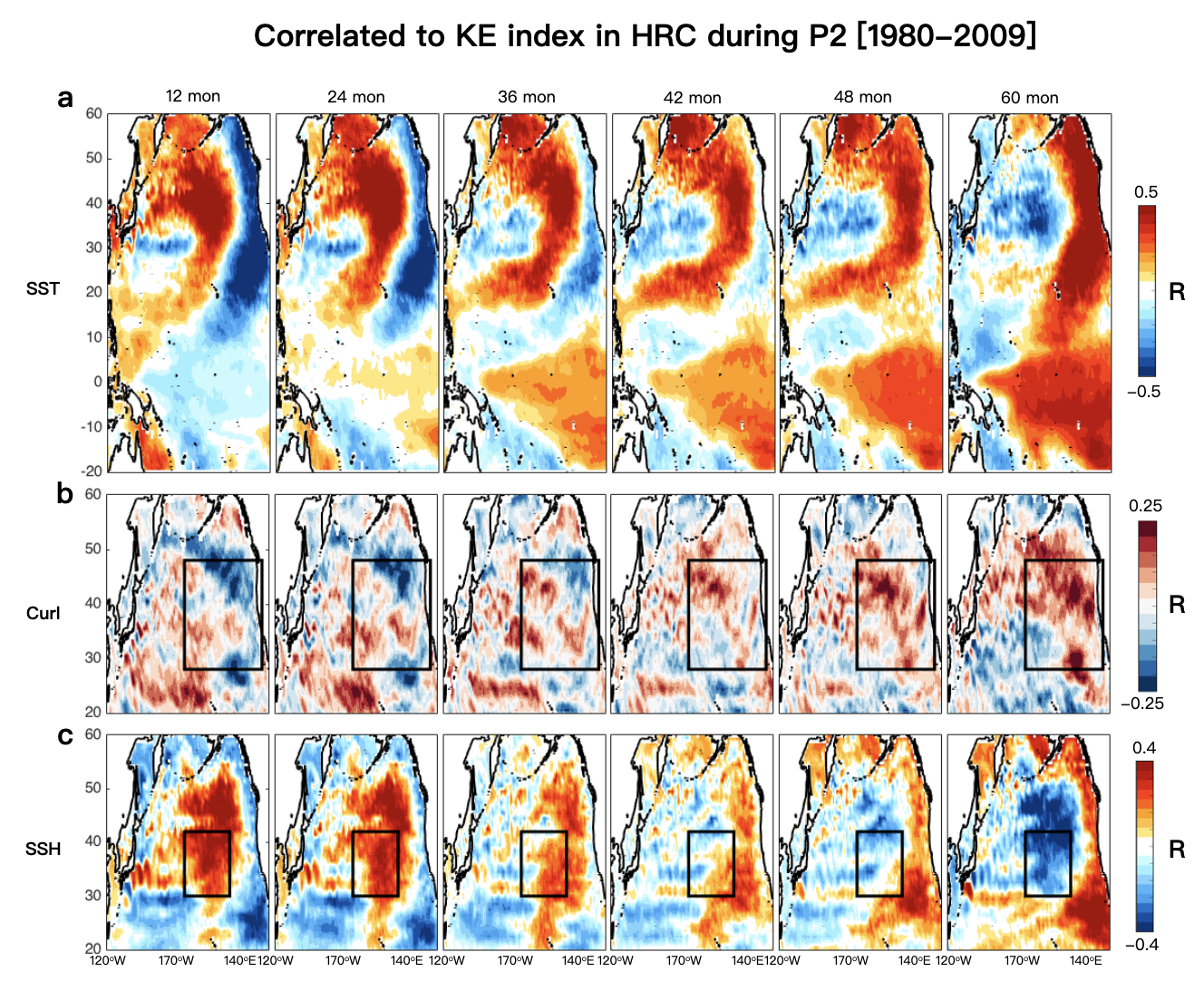
**

**Figure S1.** Spatial evolution of KE atmospheric/oceanic response. Correlation map between KE and (**a**) SST, (**b**) wind stress curl, and (c) SSH anomalies at lag 12, 24, 36, 42, 48, and 60 months simulated in HRC during the recent period 1980-2009. The figures are provided using MATLAB R2017a (https://www.mathworks.com/products/new_products/release2017a.html).


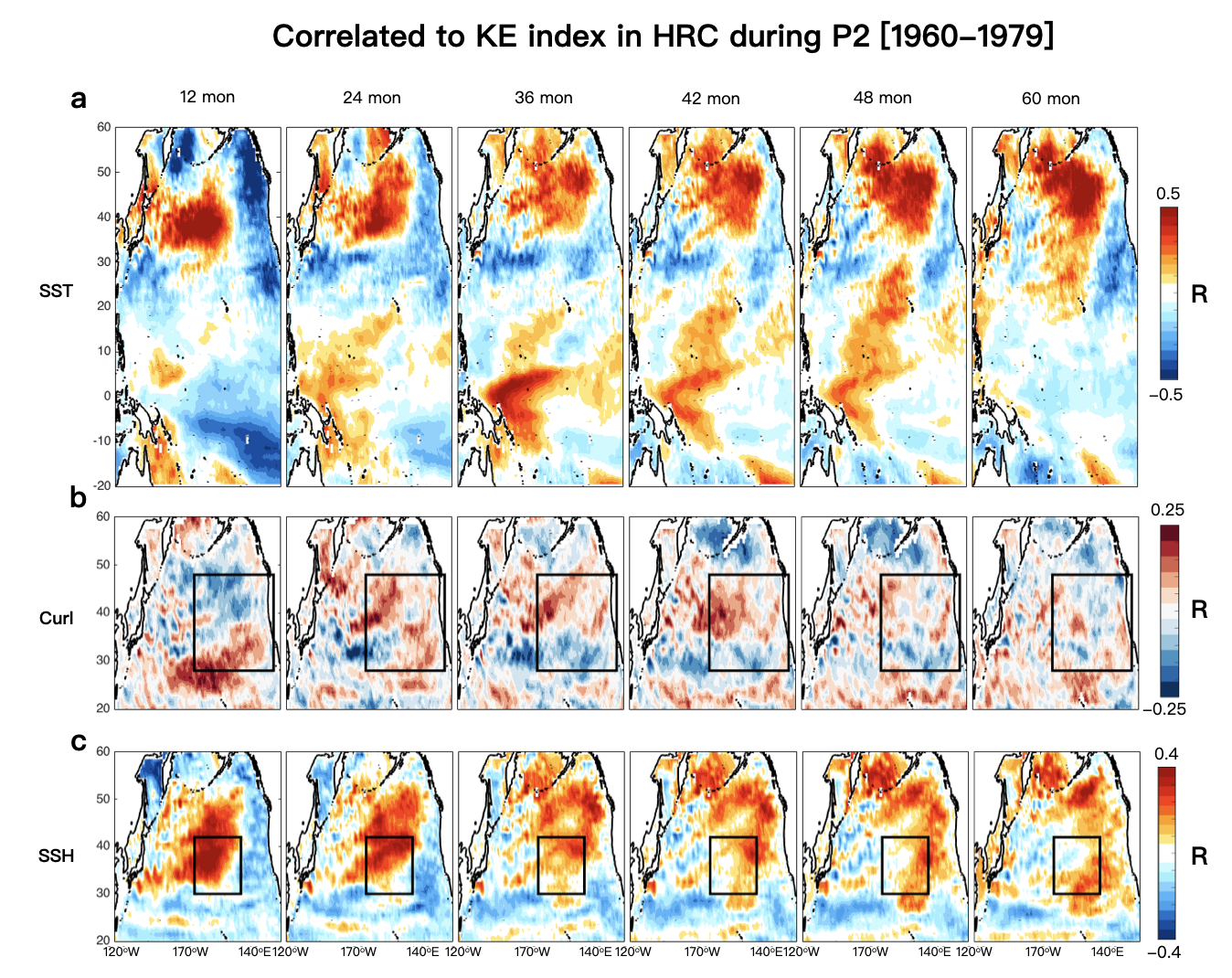


**Figure S2.** Spatial evolution of KE atmospheric/oceanic response. Correlation map between KE and (**a**) SST, (**b**) wind stress curl, and (c) SSH anomalies at lag 12, 24, 36, 42, 48, and 60 months simulated in HRC during the previous period. The figures are provided using MATLAB R2017a (https://www.mathworks.com/products/new_products/release2017a.html).

**
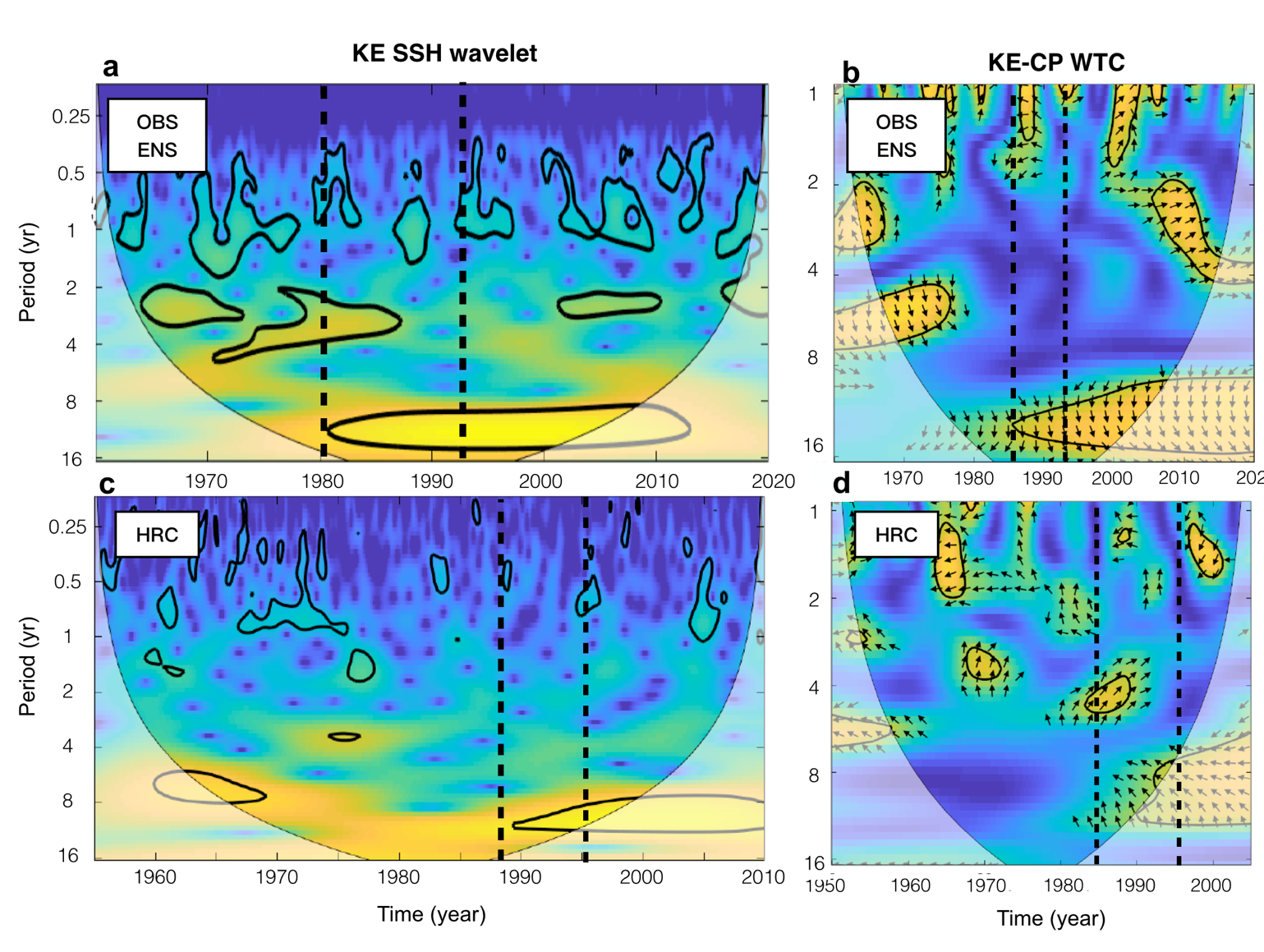
**

**Figure S3. Changes in temporal characteristics of KE and the relation between KE and CP-ENSO.** Wavelet power spectrum of the KE index (a) and wavelet transform coherence spectrum between the KE and CP-ENSO indices (b) obtained from the observations, where the relative phase relationship between two indices is denoted as arrows. Same as (c and d), but from the numerical simulations. The figures are provided using MATLAB R2017a (<https://www.mathworks.com/products/new_products/r> elease2017a.html).

**
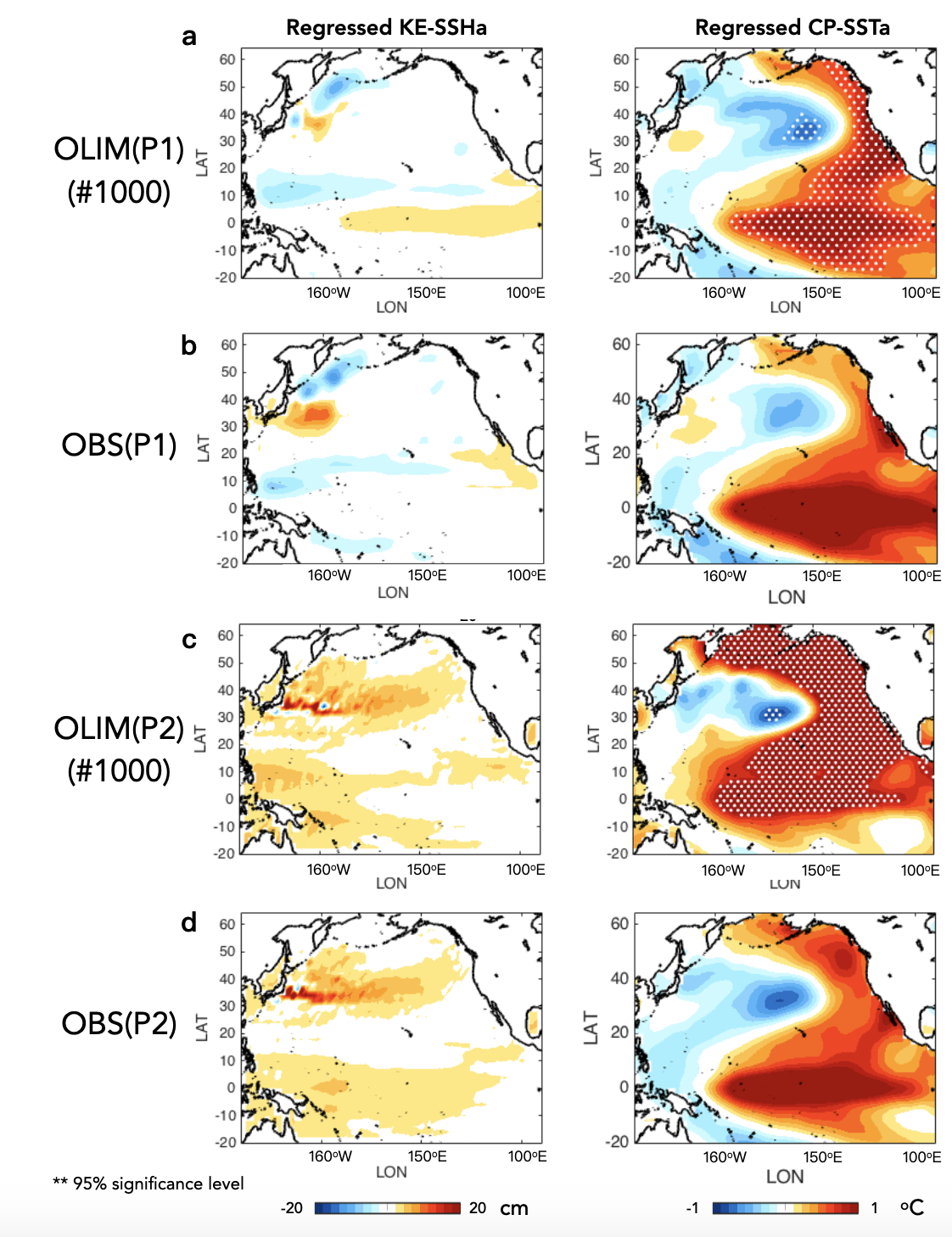
**

**Figure S4. Spatial patterns of KE and CP-ENSO in OLIM and HRC.** Regression of monthly SSH (left) and SST (right) anomalies onto the KE and CP-ENSO indices of (a) LIM ensemble mean and (b) observations for P1 and (c) LIM ensemble mean and (d) observations for P2. A dot symbol denotes the grid whose signal is significant at the 95% confidence level using a Monte-Carlo approach. The figures are provided using MATLAB R2017a (<https://www.mathworks.com/products/new_products/> release2017a.html).


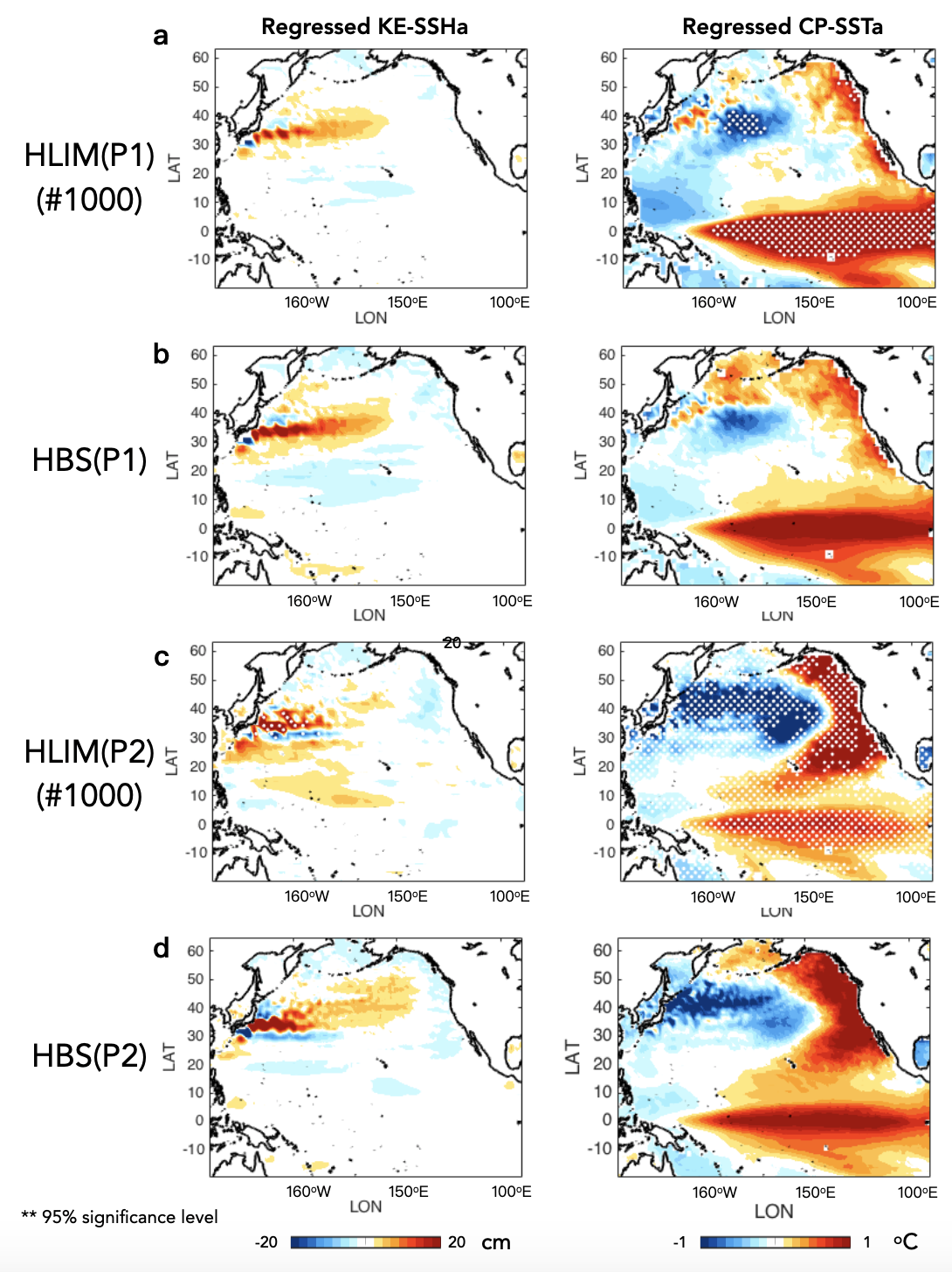


**Figure S5. Spatial patterns of KE and CP-ENSO in HLIM and HRC.** Regression of monthly SSH (left) and SST (right) anomalies onto the KE and CP-ENSO indices of (a) LIM ensemble mean and (b) HRC for P1 and (c) LIM ensemble mean and (d) HRC for P2. A dot symbol denotes the grid whose signal is significant at the 95% confidence level using a Monte-Carlo approach. The figures are provided using MATLAB R2017a (https://www.mathworks.com/products/new_products/release2017a.html).


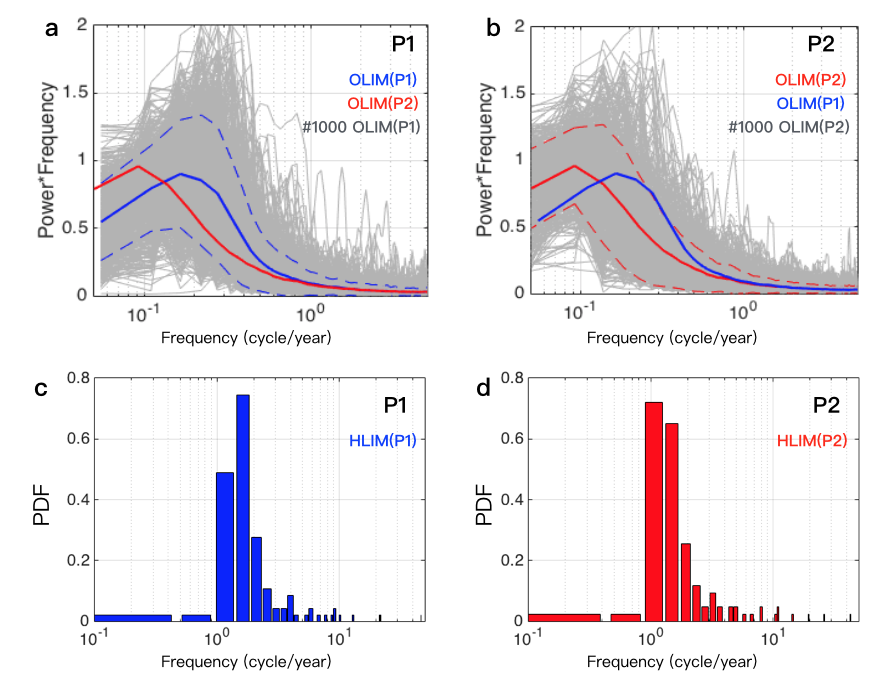


**Figure S6. Changes in KE power spectra from P1 to P2.** Spectra of the KE index for each 25-year segment (gray lines), their ensemble mean (blue for P1 and red for P2), and 10^th^ and 90^th^ percentiles (dotted red/blue line) of each spectral signal in of (a) OLIM(P1) and (b) OLIM(P2). Probability density functions of the power spectra of the KE index in (c) HLIM(P1) and (d) HLIM(P2). The figures are provided using MATLAB R2017a (https://www.mathworks.com/products/new_products/release2017a.html).


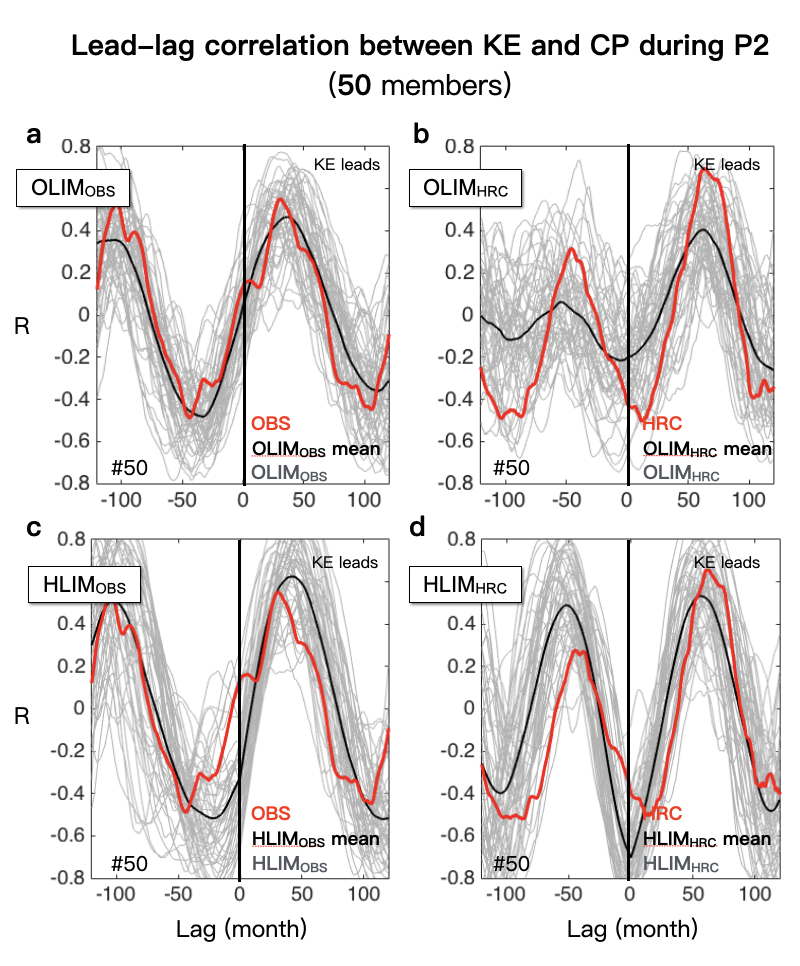


**Figure S7. Variation in the coupling between KE and CP-ENSO in the presence of stochastic noise forcing during the recent period.** Lead-lag correlation between the KE and CP-ENSO indices of subsampled 50 LIM members whose correlation is best fit to (a) the observed one and HRC’s from OLIMs. (c-d) Same as (a-b), but from HLIMs. The figures are provided using MATLAB R2017a (https://www.mathworks.com/products/new_products/release2017a.html).


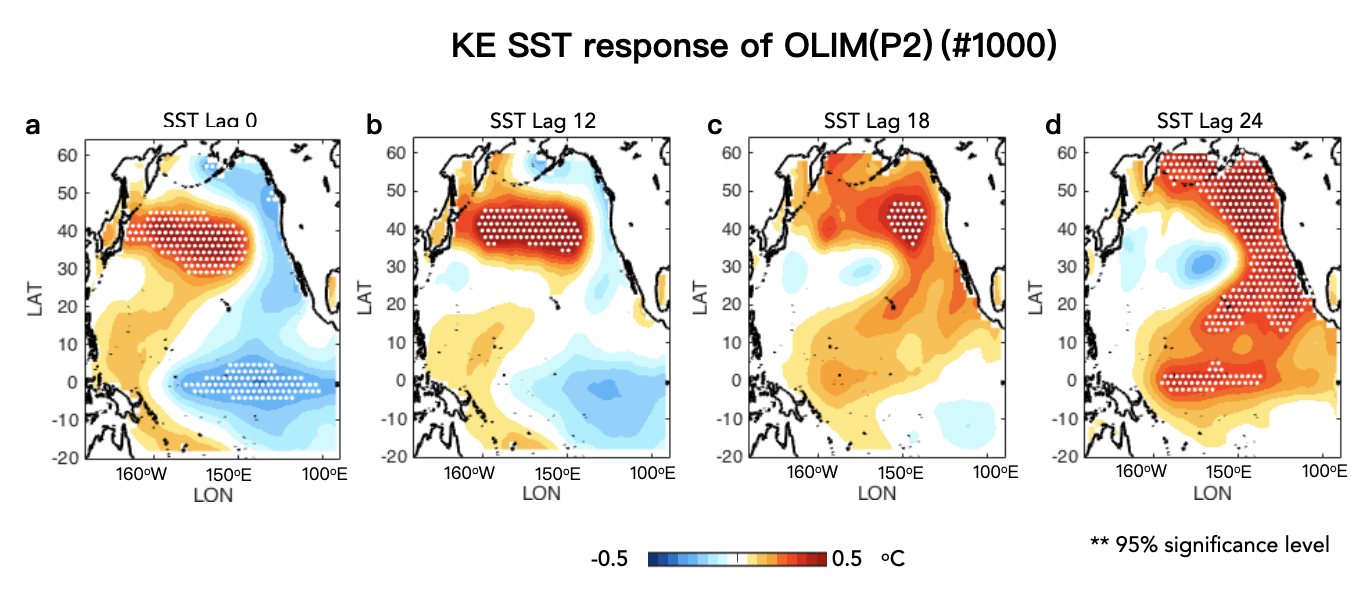


**Figure S8. Spatial evolution of KE SST in OLIM ensemble for the recent period.** LIM ensemble mean of regression between the KE index and lagged SST anomalies in each realization. A dot symbol denotes the grid whose signal is significant at the 95% confidence level in composite SST anomalies using a Monte-Carlo approach. The figures are provided using MATLAB R2017a (https://www.mathworks.com/products/new_products/release2017a.html).


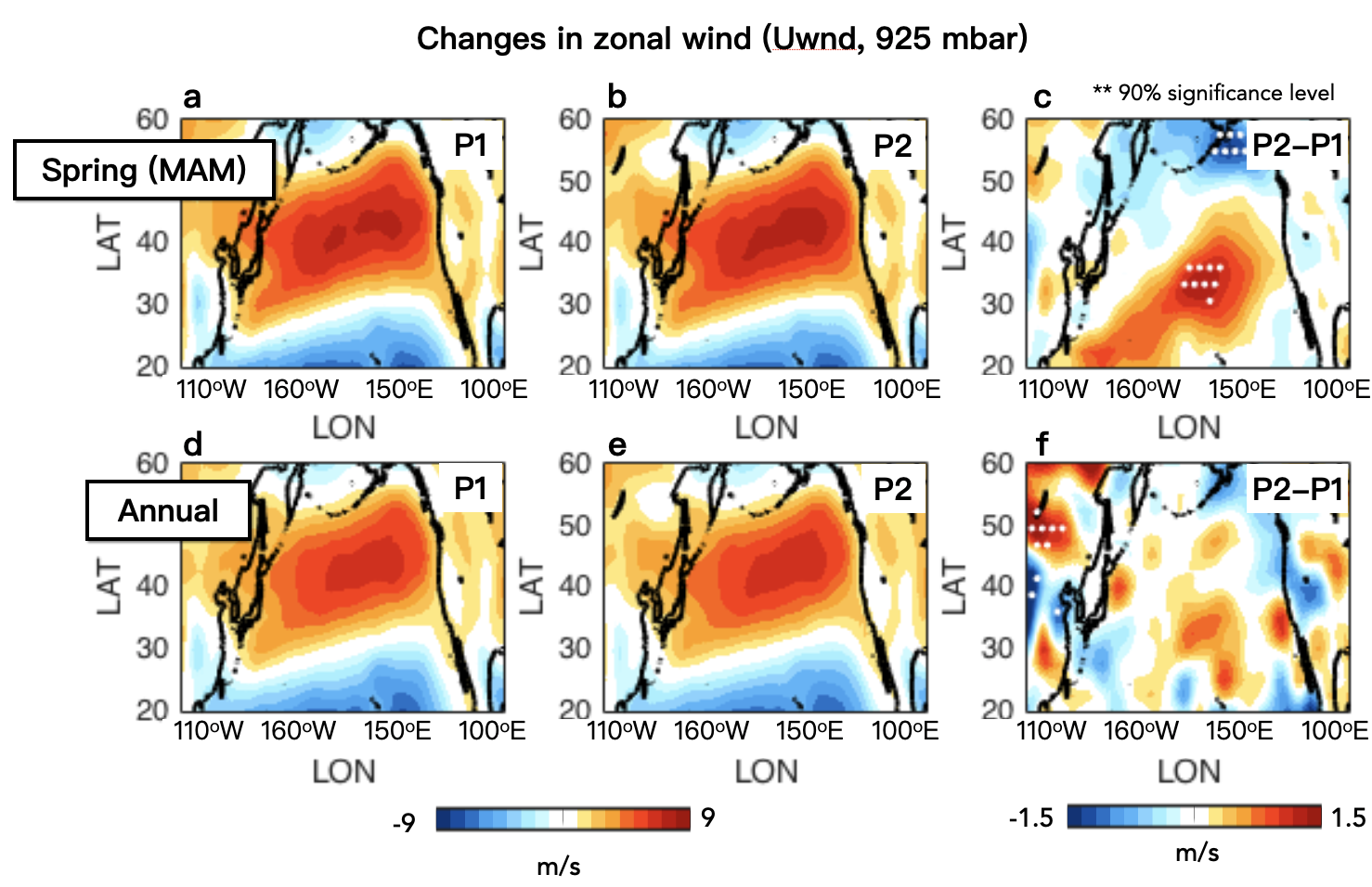


**Figure S9. Seasonality of changes in surface wind climatology.** Long-term mean of 925mb zonal wind for the spring season (March-April-May) during (a) P1 and (b) P2 and (c) their difference (P2-P1). (c-f) Same as (a-c), but for the whole season. A dot symbol denotes the grid whose signal is significant at the 90% confidence level using a Monte-Carlo approach. The figures are provided using MATLAB R2017a (https://www.mathworks.com/products/new_products/release2017a.html).
